# Aseasonal, undirected migration in insects: 'Invisible' but common

**DOI:** 10.1016/j.isci.2024.110040

**Published:** 2024-05-21

**Authors:** Thomas W. Sappington

**Affiliations:** 1 USDA, Agricultural Research Service, Corn Insects and Crop Genetics Research Unit, Ames, IA 50011, USA; 2 Department of Plant Pathology, Entomology and Microbiology, Iowa State University, Ames, IA 50011, USA

**Keywords:** environmental science, ecology, entomology

## Abstract

Many insect pests are long-distance migrants, moving from lower latitudes where they overwinter to higher latitudes in spring to exploit superabundant, but seasonally ephemeral, host crops. These seasonal long-distance migration events are relatively easy to recognize, and justifiably garner much research attention. Evidence indicates several pest species that overwinter in diapause, and thus inhabit a year-round range, also engage in migratory flight, which is somewhat "invisible" because displacement is nondirectional and terminates among conspecifics. Support for aseasonal, undirected migration is related to recognizing true migratory flight behavior, which differs fundamentally from most other kinds of flight in that it is nonappetitive. Migrating adults are not searching for resources and migratory flight is not arrested by encounters with potential resources. The population-level consequence of aseasonal, undirected migration is spatial mixing of individuals within the larger metapopulation, which has important implications for population dynamics, gene flow, pest management, and insect resistance management.

## Graphical abstract

**Figure:**
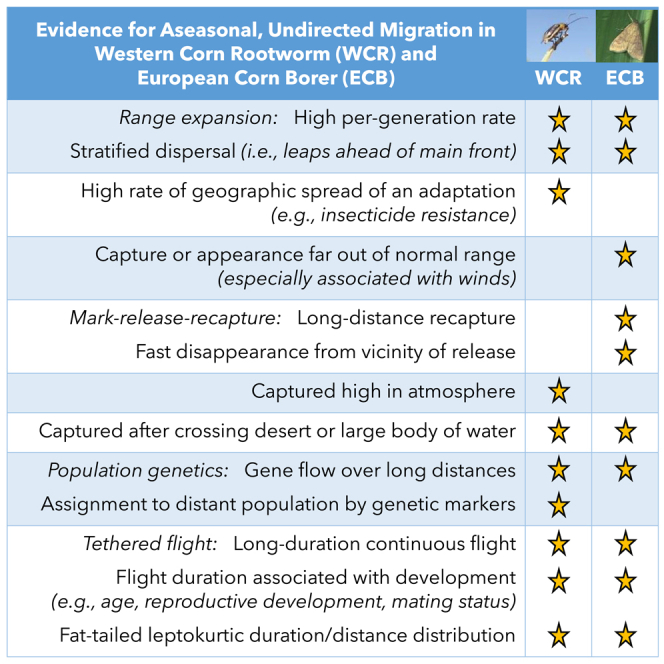

## Introduction

Insect ecologists are fully aware today that many species migrate, but this was not always so. Although it was long recognized that some insects, such as certain locusts and butterflies, could move long distances, the idea that migration was involved was, historically, a vague concept, in part because of difficulties in establishing an objective definition of migratory behavior.^1^ Beginning in the 1960s, J.S. Kennedy^1^^,^^2^ decoupled the concept of migration as a population-level phenomenon from that of migratory behavior which results in displacement. His experimentation with aphids (*Aphis fabae*) led him to see migratory flight as a specialized behavior, and he defined it in a way that allows objective experimental determination of the nature of a bout of flight activity by an insect. Familiarity with bird migration in particular had instilled the idea that true migration was round trip. Whether insects, other than diurnal butterflies, make a return migration in the fall from breeding grounds at high latitudes to the overwintering range at low latitudes was a vigorously debated question in the 1970s and 80s.^3^^,^^4^^,^^5^ Despite the short lifespans of insects usually requiring multiple generations to complete a round trip, return migration was strongly suspected based on evolutionary logic (e.g.,^6^) and indirect but compelling physiological and ecological evidence (e.g.,^7^^,^^8^^,^^9^). But it took direct evidence from radar observations^5^^,^^10^ ground-truthed with trapping data^11^ and logistically challenging, large-scale mark-release-recapture experiments accompanied by synoptic weather trajectory analyses^12^ to establish that return migrations occur. In all of these cases, the migratory movements of insects were seasonal and over long distances between distinctive overwintering and breeding regions, making them noticeable because of sudden arrivals and departures of migrants. It took additional time for the idea that migration is scale-independent to gain general acceptance, including the understanding that movement over short distances qualifies as true migration if migratory behavior is involved.^13^^,^^14^^,^^15^

Although return migration by seasonal migratory insects across long distances is now well-established,^5^ there are examples of other insect species that do not make directed migratory flights in predictable directions between seasonal ranges, but that are clearly migratory by Kennedy’s behavioral definition. In some of these species, such as the spruce budworm (*Choristoneura fumiferana*)^10^^,^^16^^,^^17^ and African armyworm (*Spodoptera exempta*),^5^^,^^10^^,^^18^ aseasonal, undirected migratory behavior seems to be an adaptation for exploiting widely scattered and unpredictable patchy habitat caused by patchy environmental conditions such as rainfall. In such cases, migration is not round trip because it does not involve directed movement between predictable seasonal habitats across latitudes or elevations, and it is easily noticed because of new outbreaks in areas previously unpopulated or sparsely populated by the species. In this article, I propose that aseasonal, undirected migration also occurs commonly in insects where breeding and overwintering ranges coincide, and where migratory behavior is not an adaptation associated with colonizing unpredictable, patchy habitat. As will become evident, the characteristics and outcomes of aseasonal, undirected migration within a year-round distribution make it somewhat "invisible," or difficult to detect, compared to seasonal, directed migration with which we are more familiar. However, the consequences of such movement are not trivial with respect to gene flow and population dynamics.

### Types of movement

In essence, Kennedy^1^^,^^2^ defined *migratory* behavior as persistent, straightened-out (non-meandering), and non-distracted movement, relative to routine day-to-day maintenance behaviors called *station-keeping.*^19^^,^^20^ Station-keeping flights are of relatively short duration as the organism forages for resources (e.g., food, mate, oviposition site, and so forth), seeks safety from a threat (e.g., escape maneuvers), or makes routine, repetitive commuting trips between habitats (e.g., for shelter or roosting) in its immediate environment. Such station-keeping flights involve a high frequency of turns resulting in a meandering flight track, so that the net displacement distance after a series of foraging flights is generally much less than the total ground-distance covered if the same effort were straightened out. Consequently, station-keeping activities of an individual occur in a relatively small area of the local landscape, defining its *home range*. Note that the home range is not necessarily anchored by a specific central place to which the individual habitually returns, such as a nest, nor does it imply territorial defense or patrolling behaviors. The home range is defined as the area of habitat over which an individual’s normal maintenance (station-keeping) activities occur. Because an individual’s home range is an emergent property of its day-to-day maintenance behaviors that involve locomotion, it is a concept that applies widely across taxa. There will be a central tendency or “site attachment” of an individual to its home range,^21^ but the dimensions of the home range can be large, small, or fluid depending on the nature of the habitat, feeding and breeding characteristics, flight capacity, and other traits of the organism.^19^^,^^20^ Net spatial displacement of multiple individuals engaged in station-keeping behavior from a specified area over a specified time interval can be described statistically at the population-level as diffusion, generating a thin-tailed Gaussian dispersal distance kernel.^22^ Because station-keeping flights are *appetitive*, the flight of a foraging insect is arrested by an encounter with the resource being sought. A migrating insect, in contrast, is engaged in *nonappetitive* flight and is not distracted by resources, regardless of how eminently suitable and attractive they might otherwise be.

*Ranging* is a major type of appetitive behavior, which in many respects can be thought of as extended foraging.^19^^,^^20^ For example, if a crop field constituting the core of an individual insect pest’s home range begins to senesce, the insect may leave this deteriorating habitat patch and search the nearby landscape for a crop at a more suitable phenological stage. The flight track of a ranging insect is usually somewhat less meandering than during station-keeping, for example as it traverses clearly unsuitable habitat in the landscape matrix. This has the effect of increasing net displacement distance compared to normal station-keeping flight within a habitat patch. The total distance flown depends on how far the individual happens to travel before finding the sought-for resource. The main difference between ranging and station-keeping is that a ranging insect moves far enough in search of a resource that it permanently leaves its former home range.^20^ Both kinds of behavior are appetitive, so the distinction between them is categorical, based on displacement distance relative to the home range. At the motivational level they are similar, both involving responses to proximate conditions (e.g., host plant quality, crowding) or immediate internal needs (e.g., hunger, need to oviposit developed eggs). Although often called “migration” based on population-level outcomes, seasonal movement between distinct reproductive and diapause (or aestivation) habitat within the same local landscape is generally accomplished via appetitive ranging flight.^23^ Examples include aphid movement between perennial overwintering hosts and herbaceous reproductive hosts;^23^ boll weevil (*Anthonomus grandis grandis*) movement between cotton fields and overwintering habitat in vegetative ground litter;^24^^,^^25^ and seasonal movement of the milkweed bug, *Lygaeus equestris*, between summer reproductive patches of milkweed and limestone outcrops where it overwinters in diapause.^15^^,^^20^

Like ranging, migratory flight takes the insect permanently out of its current home range.^19^^,^^20^^,^^26^^,^^27^ Displacement distances of long-duration ranging flights may overlap with those of short-duration migratory flights, blurring the boundary between migratory and ranging if differentiation is based on this outcome.^19^^,^^20^ However, the definition of migration is scale-independent. What matters is not distance but the pattern of the movement path and the motivation driving it.^23^^,^^28^^,^^29^ Although ranging flight can result in long displacement distances, it differs fundamentally from migratory flight in that the latter is neither triggered nor terminated in immediate response to proximate conditions or cues. Ranging flight is always facultative. Migration may be facultative, but only in the sense of being triggered by prospective cues received in an earlier, sensitive stage of development, usually a specific or narrow range of instars in larval holometabolous insects. Once the decision is made in the sensitive stage to migrate, an individual proceeds along a developmental trajectory to produce an adult exhibiting the *migration syndrome* characteristic of that particular species.^5^^,^^19^^,^^20^^,^^30^ The migration syndrome manifests as a suite of physiological, endocrine, behavioral, and sometimes morphological characters associated with migration as a process and life-history trait. Unlike ranging movement which ceases upon encountering the sought-after resource, the migrating insect is insensitive to resource cues until migratory behavior terminates in response to a global external cue such as sunrise or sunset, or an internal cue (e.g., physiological, endocrine, clock). Once migration terminates in, or over, a new location, ranging behavior begins as the insect searches for suitable habitat. When ranging movement is arrested upon encountering appropriate habitat, station-keeping behaviors resume and a new home range emerges. In some species, such as monarch butterfly, *Danaus plexxipus*, and black cutworm, *Agrotis ipsilon*, an individual may engage in migratory behavior on more than one day.^12^^,^^31^ In these cases, migratory activity usually is not continued across days without a break but can be switched on and off, and is temporally segregated within days from appetitive behaviors such as foraging for food or a roosting site.^2^^,^^20^

Facultative decisions to migrate are most obvious in *partially migratory* species, in which one portion of a population migrates (*migrants*) and the other does not (*residents*).^32^^,^^33^^,^^34^ Although fully obligate migratory species exist, partial migration is apparently the most common variety among taxa.^34^^,^^35^ Evolutionarily, partial migration is a maternal bet-hedging strategy to ensure at least some offspring survive by balancing potential costs of emigration with those of remaining to reproduce in the natal habitat. Leaving all of its offspring in the natal field or landscape has some advantages related to avoiding energy expenditure and the uncertainties of searching for suitable habitat in new environments, but risks a major loss of fitness if the habitat containing all its offspring deteriorates before the latter can escape by facultative ranging. It is a strategy that optimizes geometric rather than arithmetic mean fitness.^36^^,^^37^ The decision to migrate in a partially migratory species is probably controlled as a quantitative genetic threshold response, which serves as a developmental switch mechanism.^27^^,^^34^^,^^38^

Finally, it is important to distinguish between the movement behaviors of individuals and the consequences of such movement at either the individual or population levels. For example, the term “dispersal” is used in several different ways by ecologists, creating ambiguities in various contexts unless defined by the user.^20^^,^^21^^,^^39^ Kennedy^2^ lamented that migration and dispersal were often synonymized, and he even expressed the hope that by adopting the behavioral definition of migration the use of “dispersal” as a behavioral term would be discontinued. However, dispersal is frequently used as a synonym for movement in general^21^^,^^39^ and, at least in parts of the entomological literature, as a type of movement behavior. In this article, I define “dispersal” as the mean geographic distance between an individual’s natal site and where it leaves its offspring, following the sense of many evolutionary ecologists (e.g.,^40^^,^^41^^,^^42^). Although dispersal can be usefully defined in other ways depending on the context, the fundamental point is that it is a process and a consequence of movement behaviors, including station-keeping, ranging, and migration; it is not a type of behavior itself.^19^^,^^20^ Thus, one can speak of “dispersal distance,” “dispersal rate,” “dispersal distance kernel,” “long-distance dispersal,” and so forth because these describe consequences of movement behaviors without implying that dispersal is itself a behavior. Likewise, species that exhibit nomadism (e.g., African armyworm^5^^,^^18^) opportunistically find and exploit spatially unpredictable and ephemeral habitat patches; however, nomadism is not a behavior, but a process and life history trait. It describes a particular type of ecological and population-level outcome of undirected migratory behavior.

### Aseasonal, undirected migration

Most seasonal migrants breed actively year-round, but migrate long distances across latitudes or ecozones to track predictable spatial shifts in seasonal food supplies and environmental conditions favorable to survival and breeding.^34^ Some of these species enter reproductive diapause in preparation for and during migratory transit to overwintering regions, after which reproduction commences. A few species, such as the monarch butterfly, remain in diapause through the winter after fall migration, and others like the bogong moth (*Agrotis infusa*) migrate from winter breeding grounds to summer aestivation sites at high elevation.^43^ The focus of this article is on a type of insect migration that seems to be very common, but is mostly unrecognized or underappreciated, which I refer to here as “aseasonal, undirected migration.” This moniker emphasizes two distinguishing aspects of the behavior that differentiate it from more familiar seasonal, directed migration. First, aseasonal migration tends to terminate within the same distribution, or metapopulation, where it originates, rather than taking place between season-specific alternative regions, such as spatially distant, sometimes disjunct breeding and overwintering ranges. Thus, aseasonal migratory flight distances tend to be shorter in absolute terms than those resulting from seasonal migration across latitudes. Because it occurs within a pre-existing distribution, aseasonal migration does not change the location or spatial dimensions of the metapopulation or species distribution.

Second, the insect engaging in undirected migration has no spatial goal. The adaptive goal for aseasonal, undirected migration within a year-round distribution is not relocation to a region predictably more conducive to breeding or dormancy, but instead may be displacement itself out of the natal habitat. Such a goal is the same in principle as that of partial migration, namely spreading risk across space. Risks to the female parent’s fitness of leaving all its offspring in one habitat could include larval overcrowding, build-up of natural enemies or disease, inbreeding, or habitat destruction (e.g., by hail, flooding, fire, harvest). In the case of an aseasonal migrant moving within a year-round distribution of abundant breeding habitat, the goal of displacement can be achieved by moving in any direction and any distance beyond the natal home range. Seasonal migrants often ascend into the planetary boundary layer to take advantage of fast-moving winds which greatly enhance flight speed.^5^^,^^10^^,^^44^^,^^45^ Seasonal migrants may actively choose to ascend only into winds of favorable direction (e.g., poleward in the spring, equatorward in the fall), and many show flight orientation behavior to compensate for drift caused by winds flowing at an angle away from the preferred direction.^5^^,^^46^^,^^47^^,^^48^^,^^49^^,^^50^ In contrast, undirected migrants do not have a spatial goal, so there is no need to pick one wind direction over another or to navigate during migratory flight, except perhaps to maintain flight direction coincident with that of the wind to facilitate displacement speed or to preferentially occupy a layer of warmer air more conducive to sustained flight. An important population-level consequence of undirected migratory movement within a year-round distribution is a spatial mixing, or reshuffling, of individuals. There is no "return" migration by individuals or their descendants because there is no purposeful directionality during any migratory movement.

Pedgley^51^ listed 3 direct and 16 circumstantial types of evidence for long-distance movement by insects, which he implicitly equated with migration. Not all migration results in long-distance displacement, but most long-distance displacement (relative to station-keeping and plausible ranging distances) probably is by migration, and his list generally applies as types of evidence supporting migration as well. All three direct types of evidence are useful in detecting aseasonal, undirected migration. These include capture/recapture of marked insects, capture of insects at high altitude, and observation (e.g., via radar^10^) of individuals flying at high altitude. However, of the 16 circumstantial types, 9 are useful under normal circumstances for detecting only seasonal migration because they rely on well-defined pulses of arrivals or departures of whole populations or large numbers of individuals at a location over a short time frame. Aseasonal, undirected migration can involve large numbers of individuals moving, but will result in roughly equal rates of simultaneous immigration and emigration to and from a local area, and thus normally will not result in noticeable changes in local population density or demography. Exceptions include the detection of individuals or infestations outside the established distribution, such as during a range expansion, reintroduction to an eradication zone, or by fortuitous capture in an inhospitable environment presumably after undirected migratory flight terminated beyond the species’ habitable range.

As is true of short-distance migration,^13^^,^^14^ aseasonal, undirected movement is recognizable as true migration when it meets the definition of migratory flight behavior as described earlier: defined by Kennedy,^1^^,^^2^ elaborated by Dingle and Drake^19^ and Dingle,^20^ and widely accepted especially by insect ecologists.^5^^,^^26^^,^^27^ Aseasonal, undirected, relatively (or absolutely) short-distance movement may not match our unconscious assumptions about what migration looks like, but that does not mean it is therefore not migration. To help the reader better visualize what aseasonal, undirected migration looks like, I present case studies of two insect pests, summarizing the evidence for such migration. I focus on these two species -- western corn rootworm, *Diabrotica virgifera virgifera* (Coleoptera: Chrysomelidae) and European corn borer, *Ostrinia nubilalis* (Lepidoptera: Crambidae) – because I have conducted research related to their movement ecology and am most familiar with them. In both cases, I began my research under the unconscious assumption they are not migratory, even though I learned quickly there was considerable and incontrovertible evidence that some individuals fly long distances. This was no secret to anyone else either – Pedgley^51^ included both species in a large table listing insects for which evidence supported long distance migratory flight. But the impulse to limit "real" cases of migration to the seasonal tracking of resources, though not universal, is deep-seated.

## Case studies

### Western corn rootworm

The western corn rootworm is a major pest of corn (=maize) in North America and western Europe, causing $2-billion in losses in the U.S. annually.^52^^,^^53^ The larva feeds on roots and is the stage causing the most severe damage, although high densities of adults can occasionally disrupt pollination by feeding on corn silks. It has one generation per year, overwintering in obligate diapause as an egg in the soil.^54^ Larvae hatch in spring and feed on corn roots, passing through three instars. They pupate in the soil in mid-summer and emerge as adults. Most females mate near their emergence site.^55^^,^^56^ Although a few alternative grass species can support larval development,^57^^,^^58^ corn is by far the preferred host plant and oviposition occurs almost entirely in cornfields.^59^ The larvae cannot survive on soybean roots, making crop rotation to soybean a popular and very effective way to control this insect.^52^^,^^60^ A field planted to soybean will not receive eggs by ovipositing females, so when that field is rotated to corn the following year, no western corn rootworm larvae will be present to cause damage. An exception is found in parts of Illinois and surrounding states in the U.S. where crop-rotation resistance has evolved. Rotation-resistant females have relaxed fidelity to corn for oviposition, so soybean fields receive a portion of the eggs laid in the local landscape.^52^^,^^60^^,^^61^^,^^62^

For many farmers, planting corn in the same field for multiple years in a row ("continuous corn") is considered a better option than crop rotation for economic or other reasons.^63^^,^^64^ Any field of corn that was planted to corn the previous year is at risk of attack by rootworms. Historically, such fields were protected with a soil insecticide applied at planting. Alternatively, especially in parts of the Great Plains, aerial insecticide sprays targeting adults were used extensively to kill ovipositing females and thus protect the following year’s crop.^65^^,^^66^ Western corn rootworm has evolved resistance to several chemical insecticides.^67^ For the last 20 years, most U.S. farmers have controlled western corn rootworm by planting transgenic corn hybrids ("Bt corn") containing one or more genes from the bacterium *Bacillus thuringiensis*.^68^ These Bt genes express proteins that are toxic to rootworm larvae when they ingest root tissue, but are not toxic to adults.

To prevent or slow evolution of resistance to Bt corn hybrids, which are much more environmentally friendly than the chemical insecticides they replaced, the U.S. Environmental Protection Agency mandates that seed companies selling Bt corn implement an insect resistance management (IRM) strategy.^69^ The current IRM strategy is largely based on planting a small percentage of non-Bt corn refuge to serve as a nursery of susceptible insects to mate with any rare resistant individuals emerging from Bt corn.^68^^,^^70^ Models, experimental design, and interpretation of field data relevant to designing and evaluating IRM strategies, including containment and mitigation of resistance where it is detected, all depend on understanding western corn rootworm spatial patterns and rates of gene flow. Adult movement is the vehicle for gene flow, and thus research into all aspects of this species' movement ecology has intensified over the last two decades.

Much evidence indicates that a large proportion of western corn rootworm adults do not move very far in their lifetime. Rootworm larval populations increase year over year in fields of continuous corn,^54^^,^^71^^,^^72^^,^^73^ indicating that substantial numbers of females oviposit in the same field in which they emerged (the natal field). Resistance to Bt corn has developed independently in numerous populations in North America.^68^^,^^74^^,^^75^^,^^76^ Resistance can evolve in a single field in as few as 3 years of continuous planting of Bt corn expressing the same toxin.^74^^,^^77^ Such a rapid local response to selection is made possible by a high rate of assortative mating of resistant individuals,^64^ in turn made possible by a high rate of residency in the natal field.^68^ Movement of beetles marked by the ingestion of different types of Bt toxin^78^^,^^79^ or of ^15^N-labled corn^80^ between blocks of Bt corn and non-Bt refuge within fields, showed net displacement distances by typical station-keeping movement on the order of 20–30 m/d before mating. Trapping within cornfields showed that the abundance of adults remained higher in and near the refuge block of non-Bt corn than in the adjoining Bt corn block where larval mortality was higher and the number of adults emerging was lower. This indicated little dispersal beyond the emergence site even after mating, until later in the season as the corn matured beyond pollination. Movement between fields by ranging behavior is suggested by departure of adults from phenologically older cornfields and into phenologically younger ones.^81^^,^^82^ In addition, virtually no adults emerge from first-year cornfields after rotation from soybean, but are colonized rapidly by ovipositing females.^72^^,^^83^ Most of these adults originate from nearby fields of continuous corn,^84^^,^^85^^,^^86^ indicating local appetitive movement by ranging.

Conversely, there is a variety of equally robust evidence for long-distance migratory flight by adult western corn rootworm. Because it overwinters in diapause, any long-distance movement within the year-round distribution of the species is aseasonal. Obvious pulses of arrival and departure of large numbers are not generated from which to estimate displacement distance, indicating such flights are also undirected. However, such long-distance movement events *were* visible during this species' range expansions through North America and Europe. Western corn rootworm was originally a native of Mexico and Central America, but it spread northward, reaching current day Colorado around 1838 ce, according to genetic reconstruction.^87^ It was first reported as a regional pest of corn in Colorado in the early 1900s, and began a dramatic, well-documented eastward range expansion in the 1940s, reaching the Atlantic seaboard by the mid-1980s.^52^^,^^54^ Importantly, the spread showed a pattern of stratified dispersal:^88^^,^^89^ spread along the main front was relatively slow, depending on rates of diffusion by appetitive station-keeping and ranging flights. But it was common to see infestations far ahead of the main invasion front founded by long-distance leaps of colonizers.^90^^,^^91^^,^^92^ Distances of these leaps ranged from 20 to 200 km/year, and must be considered minimum flight distances because it almost certainly takes more than one mated female to successfully found a new population.^93^^,^^94^ Individuals moving farther but not establishing a population would not be detected. The presence of abundant habitat between the main invasion front and the new infestations strongly supports arrival of the founders by migratory flight rather than appetitive ranging flight.

The invasion history of Europe by western corn rootworm is complicated by multiple independent introductions from the U.S.,^95^^,^^96^ creating several disjunct populations from which the species spread. Nevertheless, patterns of stratified dispersal were evident,^97^^,^^98^ with distances from the nearest main invasion front ranging from 40 to 100 km.^99^^,^^100^ Population assignment techniques based on genetic markers indicated the nearest invasion front was not always the most probable source of founder populations, supporting even greater flight distances. Because founder effects often include genetic bottlenecks,^101^^,^^102^^,^^103^ these various populations were genetically differentiated across panels of selectively neutral genetic markers.^95^^,^^96^^,^^97^^,^^98^^,^^104^ Two large spreading populations with different marker profiles came in contact in northern Italy creating a zone of contact or hybrid zone, and Bermond et al.^105^ analyzed rate of clinal decay and width of zone of linkage disequilibrium among the markers to estimate dispersal distances of 13–21 km/generation. Estimates of gene flow based on classical population genetics analyses suggest little differentiation over great distances.^106^^,^^107^^,^^108^ However, they have not been useful for estimating rates of migrant exchange between locations in Europe or in the U.S. east of the Great Plains presumably because the key assumption of gene flow-genetic drift equilibrium^109^ had not yet been met since the recent invasions.

Like species range expansion, rate of spread of an adaptive trait such as insecticide or rotation resistance can provide minimum estimates of potential flight distances.^76^ Resistance to crop rotation in western corn rootworm began at a point source in Ford County, Illinois, which then spread outward in all directions over a period of years.^52^^,^^110^ A pattern of stratified dispersal is evident in maps of the spread, and expansion rate ranged from 10 to 30 km/generation against or with the prevailing winds, respectively.^111^ As in the case of the species' range expansion, this must be considered a minimum estimate of movement capacity of individuals, with the further caveat that resistance is a trait under selection adding complexities to the interpretation of spread dynamics.

Flight at high altitude is a strong indicator of migratory behavior,^45^ and capture or observation above the flight boundary layer can be considered direct evidence.^51^ Western corn rootworm were captured in light traps on a radio tower from 76 to 275 m above ground level,^112^ and numbers of adults were observed clinging to the surface of a tall building in Chicago at ∼130 m.^113^ Large numbers of adults, 89% female, found washed up on beaches of Lake Michigan during a 3-year study were associated with passage of cold fronts.^114^ The mechanism of deposition into the lake could be explained if migrants encountered a typical onshore wind while crossing the lake at 2-km elevation, as modeled by Isard et al.^115^ Ascent of large numbers of western corn rootworm into the atmosphere has been documented by netting individuals from 10-m towers raised in cornfields in Illinois.^113^^,^^116^^,^^117^ Roughly 85–90% were female,^117^ and 99% of females were mated with immature ovaries.^113^^,^^116^

As with many insects,^118^^,^^119^ tethered flight experiments of western corn rootworm from several laboratories have shown a leptokurtic distribution of uninterrupted flight durations and/or distances, with many short-duration flights and a long "fat" tail of sustained long-duration flights ranging up to 230 min.^120^^,^^121^^,^^122^^,^^123^^,^^124^^,^^125^ Although experimental conditions and beetle age-pooling varied widely between studies, the percentage of tested adults making a sustained flight of at least 20 min was greatest between 2 and 9 days old, ranging from about 20 to 50%, and was greater in females than males.

In many migratory species, the migration of females occurs early in the pre-oviposition period after emergence, a pattern called the "oogenesis-flight syndrome." While this syndrome is not universal among migratory species,^16^^,^^27^^,^^126^^,^^127^^,^^128^^,^^129^ its presence is supporting evidence for migratory behavior. Both flight mill and atmospheric ascent data are consistent with this syndrome, providing more support for migration by some female western corn rootworm.

### European corn borer

The European corn borer was introduced to northeastern North America from Europe at least three different times in the early 20th century.^130^^,^^131^ There are two pheromone races that produce and respond to different ratios of the (*E*)- or (*Z*)-11-tetradecenyl-acetate isomers comprising the female sex pheromone.^132^^,^^133^ In addition there are two voltinism races: univoltine, which has an obligate diapause, and bivoltine, in which diapause is a facultative response to photoperiod and temperature.^134^^,^^135^ Despite its name, the bivoltine race may have 1 to 4 generations per year depending on latitude, with 2 generations most common in the U.S. Corn Belt. Combinations of voltinism and pheromone system create three races,^136^ univoltine-Z (UZ), bivoltine-Z (BZ), and bivoltine-E (BE), which are partially reproductively isolated, but hybridize at low rates in areas of overlap.^137^^,^^138^^,^^139^ The species spread from its initial introduction sites in Massachusetts, New York, and Ontario throughout most corn growing regions of the U.S. and Canada east of the Rocky Mountains by the late 1970s, but the distributions of the races differ. The BZ race is found throughout the species' range in North America, the BE race is confined to the eastern quarter of the North American distribution, and UZ is found in the northern U.S. and all corn growing regions of Canada.^135^^,^^137^^,^^140^

This species is a pest of several crops and has a wide host range, but under most environmental circumstances corn is the primary host when available.^135^^,^^137^^,^^141^^,^^142^ Young larvae feed on leaves and bore into the stalk as 3rd or 4th instars, causing structural damage to the plant and disrupting nutrient flow.^143^^,^^144^ Fifth (last) instars either enter diapause to overwinter and pupate in the spring, or pupate immediately inside the stalk. A small proportion (4–18% in a study by Dalecky et al.^145^) of newly emerged females mate in the cornfield near their emergence site, but most leave the natal field before mating.^146^ Historically, European corn borer populations were consistently large throughout their range, and infestations were chronic, causing over $1-billion in losses annually.^147^ Before the commercialization of Bt corn targeting this pest in 1996, control was mainly by foliar-applied insecticides, but timing is critical to ensure exposure of larvae before they bore into the stalk where they are protected, and many farmers simply did not treat and accepted the losses.^135^ Bt corn was quickly and widely adopted in the U.S., and control was so thorough that the areawide suppression of populations has been achieved, protecting even non-Bt corn and vegetables.^148^^,^^149^ The IRM strategy, based on the high-dose/refuge concept,^150^^,^^151^ has been particularly successful for this pest, with no incidences of field-evolved practical resistance reported for the first 20 years of deployment.^152^^,^^153^ However, resistance hotspots were recently documented in several populations in four provinces of Canada to all available Bt toxins, including possible spread from the earliest hotspot in Nova Scotia to New Brunswick and Quebec.^154^^,^^155^

Slowing the evolution of resistance, its spread, and mitigation strategies all depend on understanding the movement ecology of the insect. Most flight behavior during a European corn borer’s life is appetitive, and mostly station-keeping. Adults are nocturnally active, spending the day perched in lush patches of grassy vegetation outside the cornfield in road ditches, fence lines and waterways where a humid microclimate protects them from desiccation. They tend to congregate in groups in the grass called aggregation sites.^145^^,^^156^^,^^157^^,^^158^^,^^159^^,^^160^^,^^161^^,^^162^ An exception is in semi-arid areas like the Great Plains where the moths may stay in the cornfield itself as the most humid habitat available, especially if irrigated.^163^^,^^164^^,^^165^ At dusk, moths leave the aggregation site to search for free water in the form of dew or rain droplets to imbibe.^158^ Afterward, unmated females return to the grass to release pheromone and mate, while mated females enter adjacent corn to oviposit.^156^^,^^157^^,^^159^ Most mated females return to the grass after ovipositing, but evidence suggests it is usually not the same site they occupied the previous day.^145^^,^^161^^,^^162^ Males spend most of the night casting for pheromone plumes and settle in the grass by dawn. The daily movement between habitats constitutes commuting behavior.^19^^,^^20^ Ranging behavior may be expressed if nearby grassy habitat is unsuitable,^160^ begins to deteriorate, or is destroyed by flooding or mowing. Ovipositing females have strong preferences for early planted corn in the spring and late-planted corn in the summer,^166^^,^^167^ and may search the landscape by ranging flight for a better field if the nearest cornfield is at a suboptimal phenological stage.

There is considerable evidence for European corn borer long-distance flight by migration, which is laid out in detail by Sappington,^146^^,^^168^ and summarized here. As with western corn rootworm, range expansion exhibited instances of stratified dispersal, with infestations occurring beyond the invasion front. An infestation of the UZ race in northern Ohio was most likely founded by adults flying at least 45 km over Lake Erie from Ontario.^131^ Several disjunct infestations were documented ahead of a recent expansion front as the UZ race spread north in Germany, indicating one or more long-distance leaps of 40–80 km.^169^ Rate of westward invasion by the BZ race in North America averaged 46–57 km/generation.^170^^,^^171^^,^^172^ Whether infestations are disjunct or connected to the previous position of the front, movement by founders over such distances across intervening areas with abundant, suitable host plants could only occur by non-appetitive (migratory) flight, because individuals engaged in ranging flight would be arrested by encounters with suitable habitat. Rare observations of a sudden influx of European corn borers within the year-round distribution were reported by Chiang et al.^173^ from a 240-km east-west transect of light traps along the southern border of Minnesota over two summers. Peak captures were associated with southerly winds, and they suggested that the moths were originating somewhere to the south in Iowa. Although the source was unknown and the distance need not have been very far, the data indicate a population pulse of moths moving with the wind, consistent with migratory behavior rather than events generated by ranging moths searching at ground level. Captures of European corn borer in the U.K.^174^^,^^175^^,^^176^ and southern Finland^177^ in the company of known seasonal migratory species arriving on southerly winds from the continent are indicative of migratory behavior. Pedgley and Yathom^178^ captured a European corn borer moth in southern Israel 150–300 km from the nearest possible source region, associated with a peak in capture of the beet armyworm (*Spodoptera exigua*), a well-known migratory species. Although captures in such circumstances are not definitive, it is hard to imagine a moth striking out over open water or barren desert in an appetitive search for suitable habitat. Undirected, nonappetitive migratory flight with the wind could land an individual in such places, and seems a more plausible scenario.

Observations of sudden disappearances of a large number of insects engaging in aseasonal, undirected migration within a year-round distribution are not expected for the reasons discussed earlier. However, this is routinely observed in mark-release-recapture (MRR) experiments with European corn borer (Table 1). The entomologists conducting most of these studies^145^^,^^162^^,^^163^^,^^164^^,^^179^^,^^180^^,^^181^ were careful in releasing the marked insects in a way that would not promote escape flight, and releases were made in or near attractive habitat (phenologically suitable corn, grassy field borders or plots). With a few exceptions, only a small percentage, usually less than 5% and often less than 1% of released adults were recaptured within the sampling arena (Table 1), and the departures were swift, within one or two days. The authors all concluded that a large percentage of those released must have departed the area, but expressed puzzlement because all (except^131^) had assumed most would settle nearby in attractive habitat. That was a reasonable expectation, but only for moths engaging in appetitive flight. Apparently, most of the moths were not.

**Table 1:** Summary of results of mark-release-recapture experiments on European corn borer, including rates and distances of recapture

Reference	Release habitat	No. trials	Total released	Age of released adults	Recap. method	Mean number or % recaptured per release	Range % recaptured	Range distance sampled	Max. recapture distance	Comments
Caffrey and Worthley (1927)^131^	Ocean beach	1	60,988	Most were “recently emerged” in large enclosure	SN	1 M		32 - 48 km	32 km	Daytime release on mainland, offshore wind, recapture across Cape Cod Bay
	Ocean beach	1	8,650	Unknown (feral)	SN	4 F, 3 M		1.6–16 km	8 km	Daytime release on Cape Cod, recapture along Cape
Showers et al. (2001)^179^	Open field	6 sets (of 3–5 ea.)	647,571 (males)	<24 h	PT (≥200 m) FB near release site	0.25% (per set)	0.12–0.33% (across sets)	PT: 0.2–91 km FB: 5 - 144 m	49.1 km (female in male PT)	Square rings of traps, transect distances varied over 3 years of study
Hunt et al. (2001)^163^	Irrig. corn	2	10,578	24-72 h	LT	40 F, 43 M	0.7–0.8%	3 - 207 m	207 m	Does not indicate how number released was estimated
	Non-irrig. corn	2	10,578	24-72 h	LT	6 F, 14 M	0.1–0.3%	3 - 207 m	163 m	Does not indicate how number released was estimated
Qureshi et al. (2005)^164^	Irrig. corn	7	43,019	newly emerged	PT + LT	1.05% F, 2.93% M	0.08–7.6%	15 - 1,133 m	823 m	Continuous emergence from pupae in field over multiple days; daily sampling meant those present after 1 night were detectable in principle. Only data for moths that flew beyond release point are presented here.
Reardon et al. (2006)^180^	Non-irrig. corn	12	215,900	newly emerged	SN	13 F, 90 M (0.05%)	154 to 1,580-fold fewer than expected	37 - 316 m	between 211 and 316 m	Continuous emergence from pupae in field; daily sampling meant those present after 1 night were detectable in principle
Dalecky et al. (2006)^145^	Non-irrig. corn	17	8,788	<24 h	SN	4.3%	0.2–26.9%	7 - 31 m	31 m	Recaptures were exhaustive in 100 m strip of border vegetation parallel to release site, which was 7 m into cornfield
Bailey et al. (2007)^162^	Border weeds	2	437	Unknown (feral)	SN	1.3%	0–2.6%	0 - 25 m	25 m	Recaptures were exhaustive in same 50-m strip of border vegetation where releases were made; dusk releases only, next to corn only
	Border weeds	6	2,093	Unknown (feral)	SN	7.7%	1.4–18.9%	0 - 55 m	55 m	Recaptures were exhaustive in 110-m strip of border vegetation where releases were made (in center 10-m strip); dusk releases only, next to corn only
Reardon and Sappington (2007)^181^	Small grain aggreg. plots	8	59,445	Mixed ages, 0–10 days old	SN	0.80%	0–4.4%	0 - 15 m	15 m	Recaptures from same plot of release

Irrig., irrigated; Aggreg., aggregation; SN, sweep net; PT, pheromone trap; FB, flush bar; LT, light trap; M, male; F, female.

**Figure 1:**
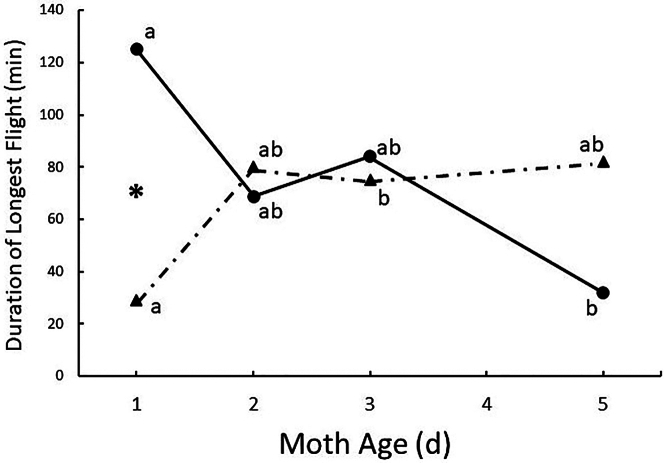
Median duration of the longest continuous flight by unmated European corn borers at different ages during 8 h of darkness on laboratory flight mills Females, circles and solid line; males, triangles and dashed line. Sample sizes per age ranged from 42 to 49 for females, and 34–47 for males. Values within sex followed by the same letter are not significantly different (α = 0.05) (Kruskal-Wallis test). ∗, significant difference between males and females at indicated age (α = 0.05) (Wilcoxon Rank-Sum Test) (From Dorhout et al. 2008.^169^).

**Figure 2:**
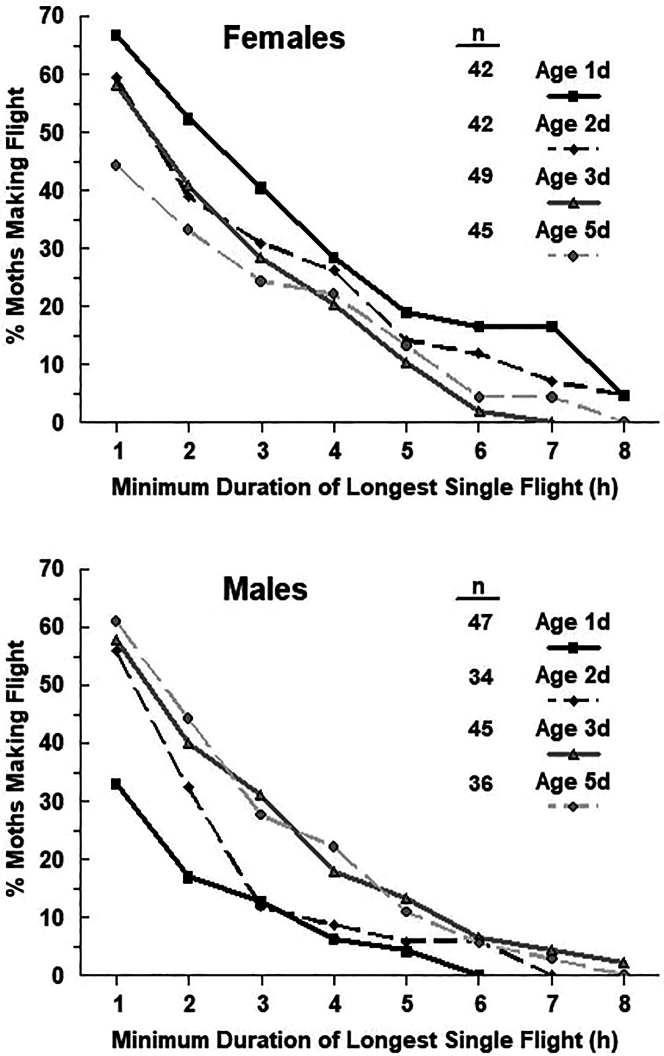
Decumulation curves showing the percentage of unmated European corn borers of different ages engaging in continuous flights on flight mills of indicated minimum durations (From Dorhout et al. 2008.^169^).

Flight performance of European corn borer adults was examined in the laboratory using rotary flight mills.^182^^,^^183^ Females tended to make their longest uninterrupted flight on the night after emergence, whereas flight duration was lowest at that age for males (Figure 1).^182^ About 17% of 1-d old females made an uninterrupted flight of at least 7 h (Figure 2).^182^ Flight propensity and duration of mated and unmated moths did not differ by age within sexes. Although long appetitive flights on a flight mill cannot be ruled out, the relatively high propensity of young unmated females to make very long flights without interruption is consistent with migratory flight and the disappearance of young females in MRR studies. The distances flown are dependent on speed, which are estimated to be about 3-fold lower for European corn borer on flight mills than in free flight, even in the absence of a tailwind.^146^ Presumably this is because of drag from friction at the flight mill pivot, attempts to steer or compensate for flight forced in a circular path, pulling the weight of the arm, and more.^184^^,^^185^^,^^186^ Net displacement distance in free flight will be much greater if wind-aided. There is indirect evidence that flight of migrating adults of European corn borer are aided by wind, both near the ground^179^^,^^187^ and probably higher in the atmosphere,^174^^,^^175^^,^^176^^,^^177^ and direct evidence for high-altitude flight by its sister species, the Asian corn borer (*Ostrinia furnicalis*).^188^^,^^189^

Young moths leaving the vicinity of their natal field in nonappetitive flight explains the simple observation that crop rotation does not protect a cornfield from European corn borer infestation enough to warrant its recommendation by research entomologists, extension personnel, or consultants.^135^^,^^190^^,^^191^ Likewise, the destruction of corn residue is very effective in killing overwintering larvae, but provides that field no protection from the following generation.^191^^,^^192^^,^^193^^,^^194^ Infestation levels in a field in one generation are simply unrelated to infestation levels in the same field the next generation.^171^^,^^195^^,^^196^ Similarly, the occupation of grassy aggregation sites by adults in the spring is related only to the adjacent current year’s crop type, not to the previous year’s crop.^161^ Grassy ditches flanked by cornfields on both sides of a road that had been planted to soybean the year before, and hence would have no overwintered European corn borer emerging from them, were just as likely to be occupied by moths at similar densities as ditches flanked by cornfields planted to corn the previous year, from which overwintered moths would be emerging. Because adults are not more abundant in grass adjacent to cornfields with emerging moths, the implication is that newly emerged adults do not preferentially occupy the first grassy ditch they encounter, but instead leave the vicinity of their natal field via nonappetitive flight.

Population genetics studies indicate low genetic differentiation and high gene flow among European corn borer populations over long distances in both the U.S.^197^^,^^198^^,^^199^^,^^200^ and Europe.^201^^,^^202^^,^^203^^,^^204^^,^^205^ Estimated distances of movement based on gene flow are at least 700 km in the U.S.^198^ and 600 km in France.^201^^,^^204^ Kim et al.^199^ used field estimates of population density and population genetics data to calculate Wright’s genetic neighborhood area.^40^^,^^206^^,^^207^^,^^208^^,^^209^ This is the area in which the parents of ∼87% of individuals at a location are expected to have originated under an assumption of diffusive movement, which follows a two-dimensional Gaussian (i.e., normal) distribution. The radius of Wright’s neighborhood area is therefore an estimate of net lifetime displacement of 87% of individuals from the natal site. For European corn borer, this radius was calculated as ∼12 km. The corollary is that ∼13% of adults move >12 km from the natal site.

### Reid’s and Slatkin’s paradoxes: western corn rootworm and European corn borer

In both the case of western corn rootworm and European corn borer, there is a wide range of estimates of lifetime dispersal. For western corn rootworm, estimates from ecological and behavioral data have demonstrated displacement distances of only a few dozen meters per day, and that many rootworms leave most or all of their offspring in their natal field. At the same time, range expansion and high altitude flight data indicate movement over long distances, up to a few hundred km.^210^ European corn borer shows the same kind of incongruence between estimates of movement.^146^^,^^168^ Short distance movement is concluded from MRR studies where only the small percentage of released individuals that remain within the sampling arena are liable to recapture and analysis, whereas long-distance movement is inferred from range expansion data and captures far from the nearest likely source. This kind of situation (i.e., conflicting evidence for displacement over short and long-distances) is known as *Reid’s paradox*,^211^^,^^212^ and is not uncommonly encountered in the ecological literature of plants.^213^^,^^214^^,^^215^^,^^216^^,^^217^ Reid’s paradox appears also to be common in insects, although not called by name, as evidenced by fat-tailed frequency distributions of flight distances (as in tethered flight data^118^^,^^119^); patterns and distances of movement, especially obvious during range expansions,^88^^,^^89^ that cannot be accounted for by a diffusion model;^6^^,^^89^ or capture of small, weak-flying insects high in the atmosphere previously assumed not to be migratory, such as *Anopheles* mosquitoes and parasitoid wasps.^218^^,^^219^ Similarly, ecological data often indicate much shorter displacement distances for a species than do estimates of gene flow from population genetics studies, a dilemma called *Slatkin’s paradox*;^109^^,^^215^^,^^216^ this paradox is evident for both western corn rootworm and European corn borer.^168^^,^^210^

The solution to these paradoxes is actually fairly simple in most cases. The problem seldom lies with faulty or inadequate methodologies giving rise to spurious conclusions when used to address the question "how far do individuals of this species move?" The real solution for a given species is almost always the most obvious: that both long and short distance flights occur, and these are generated by different movement behaviors expressed among and/or within individuals in a population.^39^^,^^215^ Different methodologies are better than others at detecting displacement within a certain range of distances, and when a variety of methodologies are employed, a variety of displacement distances may be detected – hence the apparently incongruous results that give rise to Ried’s and Slatkin’s paradoxes. But the variety of distances is real.

### Nature of evidence for aseasonal, undirected migration and limitations

The combined types of circumstantial evidence for long-distance movement via migratory behavior is robust for both western corn rootworm and European corn borer. It is perhaps most directly visualized with flight mill data, which exhibit a positively skewed, leptokurtic distribution of flight distances: most individuals during a given trial period fly only short distances while a few fly long distances. Similarly, most or all flights made by an individual are short distance, while longer flights are comparatively rare. This is a common result for almost any insect,^119^^,^^220^^,^^221^^,^^222^^,^^223^ whether it is migratory or not. But migratory flight is suggested by the shape of the distribution’s tail. The positively skewed distribution of flight distance (or duration) generated by only appetitive flight will display a "skinny" tail, showing a sharp drop off in the frequency of long flights to zero.^222^^,^^223^ If some individuals in the trial population engage in migratory flight, the distribution will display a "fat tail" with a more gradual decline in the frequency of long flights. Both western corn rootworm and European corn borer show a fat-tailed distribution on flight mills, indicating migratory flight behavior.^168^^,^^210^ It is presumed that the overall distribution of flight distance is generated by the superimposing of two different dispersal distance kernels^22^ exhibited in the population: short-range appetitive flight with a skinny tail and the mode frequency not far from zero along the x axis, and long-range migratory flight where mode frequency is shifted to the right followed by a long, fat tail.^212^^,^^213^^,^^223^

Evidence clearly supports the concept that these two species are partially migratory. Distinct portions of the population that either never migrate (residents) or do migrate (migrants) are inferred for both.^146^^,^^168^^,^^182^^,^^210^ In the case of the western corn rootworm, a very large proportion (at least half, maybe closer to three-quarters) are residents that engage only in appetitive flight behavior (station-keeping, ranging) throughout adulthood, while a smaller but substantial proportion make at least one migratory flight.^210^ Conversely, a large proportion (perhaps 90%) of European corn borer adults migrate soon after emergence, although a small proportion do not and settle in the vicinity of the emergence site.^168^

If taken alone, each line of evidence for either species could perhaps be plausibly attributed to other causes or to appetitive behavior. For example, there are many well-known pitfalls in interpreting MRR data. Although recapture of a marked insect indicates definitively a minimum distance flown over the time period since release, MRR experiments are not good at providing information on maximum flight distances because of the dilution of marked-insect density with distance from the release site.^224^^,^^225^ There is also a potential problem of adult crowding at the release site contributing to a proximate facultative decision to emigrate rapidly by behavior unrelated to migration. It can be challenging to distinguish long-distance ranging from migratory flight in flight mill studies, which may over or underestimate flight durations because of a tethered insect’s inability to control its direction or to fly in three dimensions. Fundamentally, it is usually very difficult to definitively discern an individual’s immediate motivation for flight. Our ability to determine whether a flight is appetitive or nonappetitive is often limited by incomplete knowledge regarding the specific cues the insect uses and/or is exposed to which might arrest flight when encountered. Such knowledge is necessary when relying on Kennedy’s^1^^,^^2^ behavioral definition to conclusively detect migratory flight. While multiple lines of indirect evidence all pointing to migratory behavior can be compelling, as in the cases of western corn rootworm and European corn borer described above, it is important to keep these limitations in mind.

## How common is aseasonal, undirected migration?

It is likely that aseasonal, undirected migration is common among other species. Sweet potato whitefly (*Bemesia tabaci*), was recognized as this type of migrant when tiny size and short migratory distances made it a hard sell at the time.^13^^,^^226^ Boll weevil (*Anthonomus grandis grandis*), which overwinters in-place in diapause,^227^ is an aseasonal, undirected migratory species. Evidence of boll weevil migratory movement up to hundreds of km includes rates of range expansion,^228^ reintroductions to eradication zones associated with wind events,^229^^,^^230^ recapture of marked weevils many km from the release site,^231^^,^^232^ captures of weevils far from the nearest possible source,^233^^,^^234^^,^^235^ capture at high altitude,^236^^,^^237^ estimates of gene flow between locations,^238^^,^^239^ and immigrant identification and population assignment to the most likely source using genetic markers.^229^^,^^240^^,^^241^ Other likely candidates with various lines of evidence include brown marmorated stink bug (*Halyomorpha halys*),^242^^,^^243^ mountain pine beetle (*Dendroctonus ponderosae*),^244^^,^^245^ spruce budworm,^16^^,^^17^ and navel orangeworm (*Amyelois transitella*).^246^ While somewhat invisible compared to seasonal, directed migration, the manifestation of Reid’s or Slatkin’s paradox may constitute a signature of aseasonal, undirected migratory behavior that has heretofore gone unrecognized.

## References

[1] Kennedy J.S. (1961). A turning point in the study of insect migration. Nature.

[2] Kennedy J.S., Rankin M.A. (1985). Migration: Mechanisms and Adaptive Significance.

[3] Walker T.J. (1980). Migrating Lepidoptera: Are butterflies better than moths?. Fla. Entomol..

[4] Stinner R.E., Barfield C.S., Stimac J.L., Dohse L. (1983). Dispersal and movement of insect pests. Annu. Rev. Entomol..

[5] Chapman J.W., Reynolds D.R., Wilson K. (2015). Long-range seasonal migration in insects: mechanisms, evolutionary drivers and ecological consequences. Ecol. Lett..

[6] Dingle H. (1982). Function of migration in the seasonal synchronization of insects. Entomol. Exp. Appl..

[7] Kaster L.V., Showers W.B. (1982). Evidence of spring immigration and autumn reproductive diapause of the adult black cutworm in Iowa. Environ. Entomol..

[8] Turgeon J.J., McNeil J.N. (1983). Modification in the calling behavior of *Pseudaletia unipuncta* (Lepidoptera: Noctuidae), induced by temperature conditions during pupal and adult development. Can. Entomol..

[9] McNeil J.N. (1987). The true armyworm, *Pseudaletia unipuncta*: a victim of the Pied Piper or a seasonal migrant?. Int. J. Trop. Insect Sci..

[10] Drake V.A., Reynolds D.R. (2012).

[11] Pair S.D., Raulston J.R., Rummel D.R., Westbrook J.K., Wolf W.W., Sparks A.N., Schuster M.F. (1987). Development and production of corn earworm and fall armyworm in the Texas high plains: evidence for reverse fall migration. Southwest. Entomol..

[12] Showers W.B., Keaster A.J., Raulston J.R., Hendrix W.H., Derrick M.E., McCorcle M.D., Robinson J.F., Way M.O., Wallendorf M.J., Goodenough J.L. (1993). Mechanism of southward migration of a noctuid moth [*Agrotis ipsilon* (Hufnagel)]: a complete migrant. Ecology.

[13] Byrne D. (1999). Migration and dispersal by the sweet potato whitefly, *Bemisia tabaci*. Agric. For. Meteorol..

[14] Byrne D.N., Koul O., Cuperus G., Elliott N. (2008). Areawide Pest Management: Theory and Implementation.

[15] Solbreck C., Rankin M.A. (1985). Migration: Mechanisms and Adaptive Significance.

[16] Greenbank D.O., Schaefer G.W., Rainey R.C. (1980). Spruce budworm (Lepidoptera: Tortricidae) moth flight and dispersal: new understanding from canopy observations, radar, and aircraft. Mem. Entomol. Soc. Can..

[17] Sturtevant B.R., Achtemeier G.L., Charney J.J., Anderson D.P., Cooke B.J., Townsend P.A. (2013). Long-distance dispersal of spruce budworm (*Choristoneura fumiferana* Clemens) in Minnesota (USA) and Ontario (Canada) via the atmospheric pathway. Agric. For. Meteorol..

[18] Rose D.J.W., Dewhurst C.F., Page W.W. (2000).

[19] Dingle H. (2014).

[20] Dingle H., Drake V.A. (2007). What is migration?. Bioscience.

[21] Roshier D., Reid J. (2003). On animal distributions in dynamic landscapes. Ecography.

[22] Nathan R., Klein E., Robedo-Arnuncio J.J., Revilla E., Clobert J., Baguette M., Benton T.G., Bullock J.M. (2012). Dispersal Ecology and Evolution.

[23] Irwin M.E., Kampmeier G.E., Weisser W.W., van Emden H.F., Harrington R. (2007). Aphids as Crop Pests.

[24] Slosser J.E., Boring E.P. (1980). Shelterbelts and boll weevils: a control strategy based on management of overwintering habitat. Environ. Entomol..

[25] Showler A.T. (2006). Short-range dispersal and overwintering habitats of boll weevils (Coleoptera: Curculionidae) during and after harvest in the subtropics. J. Econ. Entomol..

[26] Asplen M.K. (2018). Dispersal strategies in terrestrial insects. Curr. Opin. Insect Sci..

[27] Asplen M.K. (2020). Proximate drivers of migration and dispersal in wing-monomorphic insects. Insects.

[28] Irwin M. (1999). Implications of movement in developing and deploying integrated pest management strategies. Agric. For. Meteorol..

[29] Nathan R., Getz W.M., Revilla E., Holyoak M., Kadmon R., Saltz D., Smouse P.E. (2008). A movement ecology paradigm for unifying organismal movement research. Proc. Natl. Acad. Sci. USA.

[30] Drake V.A., Gatehouse A.G., Farrow R.A., Drake V.A., Gatehouse A.G. (1995). Insect Migration: Tracking Resources Through Space and Time.

[31] Brower L.P., Fink L.S., Walford P. (2006). Fueling the fall migration of the monarch butterfly. Integr. Comp. Biol..

[32] Chapman B.B., Brönmark C., Nilsson J.-Å., Hansson L.-A. (2011). Partial migration: an introduction. Oikos.

[33] Chapman B.B., Brönmark C., Nilsson J.-Å., Hansson L.-A. (2011). The ecology and evolution of partial migration. Oikos.

[34] Menz M.H.M., Reynolds D.R., Gao B., Hu G., Chapman J.W., Wotton K.R. (2019). Mechanisms and consequences of partial migration in insects. Front. Ecol. Evol..

[35] Dällenbach L.J., Glauser A., Lim K.S., Chapman J.W., Menz M.H.M. (2018). Higher flight activity in the offspring of migrants compared to residents in a migratory insect. Proc. Biol. Sci..

[36] Philippi T., Seger J. (1989). Hedging one’s evolutionary bets, revisited. Trends Ecol. Evol..

[37] Hidalgo J., Casas R.R.d., Muñoz M.Á. (2016). Environmental unpredictability and inbreeding depression select for mixed dispersal syndromes. BMC Evol. Biol..

[38] Pulido F. (2011). Evolutionary genetics of partial migration – the threshold model of migration revis(it)ed. Oikos.

[39] Peniston J.H., Backus G.A., Baskett M.L., Fletcher R.J., Holt R.D. (2024). Ecological and evolutionary consequences of temporal variation in dispersal. Ecography.

[40] Bradburd G.S., Ralph P.L. (2019). Spatial population genetics: it's about time. Annu. Rev. Ecol. Evol. Syst..

[41] Richardson J.L., Urban M.C., Bolnick D.I., Skelly D.K. (2014). Microgeographic adaptation and the spatial scale of evolution. Trends Ecol. Evol..

[42] De Meeûs T., Ravel S., Solano P., Bouyer J. (2019). Negative density-dependent dispersal in tsetse flies: a risk for control campaigns?. Trends Parasitol..

[43] Lownds R.M., Turbill C., White T.E., Umbers K.D. (2023). The impact of elevated aestivation temperatures on the behaviour of bogong moths (*Agrotis infusa*). J. Therm. Biol..

[44] Chapman J.W., Drake V.A., Reynolds D.R. (2011). Recent insights from radar studies of insect flight. Annu. Rev. Entomol..

[45] Reynolds D.R., Chapman J.W., Drake V.A., Chilson P.B., Frick W.F., Kelly J.F., Liechti F. (2017). Aeroecology.

[46] Chapman J.W., Reynolds D.R., Mouritsen H., Hill J.K., Riley J.R., Sivell D., Smith A.D., Woiwod I.P. (2008). Wind selection and drift compensation optimize migratory pathways in a high-flying moth. Curr. Biol..

[47] Chapman J.W., Reynolds D.R., Hill J.K., Sivell D., Smith A.D., Woiwod I.P. (2008). A seasonal switch in compass orientation in a high-flying migrant moth. Curr. Biol..

[48] Chapman J.W., Nesbit R.L., Burgin L.E., Reynolds D.R., Smith A.D., Middleton D.R., Hill J.K. (2010). Flight orientation behaviors promote optimal migration trajectories in high-flying insects. Science.

[49] Chapman J.W., Klaassen R.H.G., Drake V.A., Fossette S., Hays G.C., Metcalfe J.D., Reynolds A.M., Reynolds D.R., Alerstam T. (2011). Animal orientation strategies for movement in flows. Curr. Biol..

[50] Reynolds A.M., Reynolds D.R., Sane S.P., Hu G., Chapman J.W. (2016). Orientation in high-flying migrant insects in relation to flows: Mechanisms and strategies. Phil. Trans. R. Soc. B.

[51] Pedgley D.E., Ruberson J.R. (1999). Handbook of Pest Management.

[52] Gray M.E., Sappington T.W., Miller N.J., Moeser J., Bohn M.O. (2009). Adaptation and invasiveness of western corn rootworm: intensifying research on a worsening pest. Annu. Rev. Entomol..

[53] Wechsler S., Smith D. (2018). Has resistance taken root in US corn fields? Demand for insect control. Am. J. Agric. Econ..

[54] Meinke L.J., Sappington T.W., Onstad D.W., Guillemaud T., Miller N.J., Komáromi J., Levay N., Furlan L., Kiss J., Toth F. (2009). Western corn rootworm (*Diabrotica virgifera virgifera* LeConte) population dynamics. Agric. For. Entomol..

[55] Lew A.C., Ball H.J. (1979). The mating behavior of the western corn rootworm *Diabrotica virgifera virgifera* (Coleoptera: Chrysome-lidae). Ann. Entomol. Soc. Am..

[56] Marquardt P.T., Krupke C.H. (2009). Dispersal and mating behavior of *Diabrotica virgifera virgifera* (Coleoptera: Chrysomelidae) in Bt cornfields. Environ. Entomol..

[57] Clark T.L., Hibbard B.E. (2004). Comparison of non-maize hosts to support western corn rootworm (Coleoptera: Chrysomelidae) larval biology. Environ. Entomol..

[58] Oyediran I.O., Hibbard B.E., Clark T.L. (2004). Prairie grasses as hosts of the western corn rootworm (Coleoptera: Chrysomelidae). Environ. Entomol..

[59] Bernal J.S., Medina R.F. (2018). Agriculture sows pests: how crop domestication, host shifts, and agricultural intensification can create insect pests from herbivores. Curr. Opin. Insect Sci..

[60] Spencer J.L., Hibbard B.E., Moeser J., Onstad D.W. (2009). Behaviour and ecology of the western corn rootworm (*Diabrotica virgifera virgifera* LeConte) (Coleoptera: Chrysomelidae). Agric. For. Entomol..

[61] Spencer J.L., Isard S.A., Levine E. (1999). Free flight of western corn rootworm (Coleoptera: Chrysomelidae) to corn and soybean plants in a walk-in wind tunnel. J. Econ. Entomol..

[62] Levine E., Spencer J.L., Isard S.A., Onstad D.W., Gray M.E. (2002). Adaptation of the western corn rootworm to crop rotation: evolution of a new strain in response to a management practice. Am. Entomol..

[63] Sappington T.W., Peshin R., Pimental D. (2014).

[64] Andow D.A., Pueppke S.G., Schaafsma A.W., Gassmann A.J., Sappington T.W., Meinke L.J., Mitchell P.D., Hurley T.M., Hellmich R.L., Porter R.P. (2016). Early detection and mitigation of resistance to Bt maize by western corn rootworm (Coleoptera: Chrysomelidae). J. Econ. Entomol..

[65] Pruess K.P., Witkowski J.F., Raun E.S. (1974). Population suppression of western corn rootworm by adult control with ULV malathion. J. Econ. Entomol..

[66] Meinke L.J., Siegfried B.D., Wright R.J., Chandler L.D. (1998). Adult susceptibility of Nebraska western corn rootworm (Coleoptera: Chrysomelidae) populations to selected insecticides. J. Econ. Entomol..

[67] Meinke L.J., Souza D., Siegfried B.D. (2021). The use of insecticides to manage the western corn rootworm, *Diabrotica virgifera virgifera*, LeConte: history, field-evolved resistance, and associated mechanisms. Insects.

[68] Gassmann A.J. (2021). Resistance to Bt maize by western corn rootworm: effects of pest biology, the pest-crop interaction and the agricultural landscape on resistance. Insects.

[69] USEPA (2021). Insect resistance management for Bt plant-incorporated protectants. https://www.epa.gov/regulation-biotechnology-under-tscaand-fifra/insect-resistance-management-bt-plant-incorporated.

[70] Smith E.M., Shrestha R.B., Gassmann A.J. (2023). Inheritance and fitness costs of laboratory-selected resistance to Gpp34/Tpp35Ab1 corn in western corn rootworm (Coleoptera: Chrysomelidae). J. Econ. Entomol..

[71] Hill R.E., Mayo Z.B. (1980). Distribution and abundance of corn rootworm species as influenced by topography and crop rotation in eastern Nebraska. Environ. Entomol..

[72] Sivcev I., Stankovic S., Kostic M., Lakic N., Popovic Z. (2009). Population density of *Diabrotica virgifera virgifera* LeConte beetles in Serbian first year and continuous maize fields. J. Appl. Entomol..

[73] Reinders J.D., Hitt B.D., Stroup W.W., French B.W., Meinke L.J. (2018). Spatial variation in western corn rootworm (Coleoptera: Chrysomelidae) susceptibility to Cry3 toxins in Nebraska. PLoS One.

[74] Gassmann A.J., Petzold-Maxwell J.L., Clifton E.H., Dunbar M.W., Hoffmann A.M., Ingber D.A., Keweshan R.S. (2014). Field-evolved resistance by western corn rootworm to multiple *Bacillus thuringiensis* toxins in transgenic maize. Proc. Natl. Acad. Sci. USA.

[75] Wangila D.S., Gassmann A.J., Petzold-Maxwell J.L., French B.W., Meinke L.J. (2015). Susceptibility of Nebraska western corn rootworm (Coleoptera: Chrysomelidae) populations to Bt corn events. J. Econ. Entomol..

[76] Miller N.J., Sappington T.W. (2017). Role of dispersal in resistance evolution and spread. Curr. Opin. Insect Sci..

[77] Shrestha R.B., Dunbar M.W., French B.W., Gassmann A.J. (2018). Effects of field history on resistance to Bt maize by western corn rootworm, *Diabrotica virgifera virgifera* LeConte (Coleoptera: Chrysomelidae). PLoS One.

[78] Spencer J., Onstad D., Krupke C., Hughson S., Pan Z., Stanley B., Flexner L. (2013). Isolated females and limited males: evolution of insect resistance in structured landscapes. Entomol. Exp. Appl..

[79] Hughson S.A. (2017). http://hdl.handle.net/2142/98361.

[80] Taylor S., Krupke C. (2018). Measuring rootworm refuge function: *Diabrotica virgifera virgifera* emergence and mating in seed blend and strip refuges for *Bacillus thuringiensis* (Bt) maize. Pest Manag. Sci..

[81] Hill R.E., Mayo Z.B. (1974). Trap-corn to control corn rootworms. J. Econ. Entomol..

[82] Pierce C.M.F., Gray M.E. (2006). Western corn rootworm, *Diabrotica virgifera virgifera* LeConte (Coleoptera: Chrysomelidae), oviposition: a variant’s response to maize phenology. Environ. Entomol..

[83] Godfrey L.D., Turpin F.T. (1983). Comparison of western corn rootworm (Coleoptera: Chrysomelidae) adult populations and economic thresholds in first-year and continuous cornfields. J. Econ. Entomol..

[84] Beckler A.A., French B.W., Chandler L.D. (2004). Characterization of western corn rootworm (Coleoptera: Chrysomelidae) population dynamics in relation to landscape attributes. Agric. For. Entomol..

[85] Szalai M., Kőszegi J., Toepfer S., Kiss J. (2011). Colonisation of first-year maize fields by western corn rootworm (*Diabrotica virgifera virgifera* LeConte) from adjacent infested maize fields. Acta Phytopathol. Entomol. Hung..

[86] Levay N., Terpo I., Kiss J., Toepfer S. (2015). Quantifying inter-field movements of the western corn rootworm (*Diabrotica virgifera virgifera* LeConte) – a central European field study. Cereal Res. Commun..

[87] Lombaert E., Ciosi M., Miller N.J., Sappington T.W., Blin A., Guillemaud T. (2018). Colonization history of the western corn rootworm (*Diabrotica virgifera virgifera*) in North America: insights from random forest ABC using microsatellite data. Biol. Invasions.

[88] Shigesada N., Kawasaki K., Takeda Y. (1995). Modeling stratified diffusion in biological invasions. Am. Nat..

[89] Liebhold A.M., Tobin P.C. (2008). Population ecology of insect invasions and their management. Annu. Rev. Entomol..

[90] Chiang H.C., Flaskerd R.G. (1969). Northern and western corn rootworms in Minnesota. J. Minn. Acad. Sci..

[91] Ruppel R.F. (1975). Dispersal of western corn rootworm, *Diabrotica virgifera* Le Conte, in Michigan (Coleoptera: Chrysomelidae). J. Kan. Entomol. Soc..

[92] Youngman R.R., Day E.R. (1993). Incidence of western corn-rootworm beetles (Coleoptera, Chrysomelidae) on corn in Virginia from 1987 to 1992. J. Entomol. Sci..

[93] Blackburn T.M., Lockwood J.L., Cassey P. (2015). The influence of numbers on invasion success. Mol. Ecol..

[94] Saccaggi D.L., Wilson J.R.U., Terblanche J.S. (2021). Propagule pressure helps overcome adverse environmental conditions during population establishment. Curr. Res. Insect Sci..

[95] Miller N., Estoup A., Toepfer S., Bourguet D., Lapchin L., Derridj S., Kim K.S., Reynaud P., Furlan L., Guillemaud T. (2005). Multiple transatlantic introductions of the western corn rootworm. Science.

[96] Ciosi M., Miller N.J., Kim K.S., Giordano R., Estoup A., Guillemaud T. (2008). Invasion of Europe by the western corn rootworm, *Diabrotica virgifera virgifera*: multiple transatlantic introductions with various reductions of genetic diversity. Mol. Ecol..

[97] Ciosi M., Miller N.J., Toepfer S., Estoup A., Guillemaud T. (2011). Stratified dispersal and increasing genetic variation during the invasion of Central Europe by the western corn rootworm, *Diabrotica virgifera virgifera*. Evol. Appl..

[98] Bermond G., Ciosi M., Lombaert E., Blin A., Boriani M., Furlan L., Toepfer S., Guillemaud T. (2012). Secondary contact and admixture between independently invading populations of the western corn rootworm, *Diabrotica virgifera virgifera* in Europe. PLoS One.

[99] Baufeld P., Enzian S. (2001). Proceedings XXI IWGO Conference and VIII Diabrotica Subgroup Meeting.

[100] Mrganić M., Bažok R., Mikac K.M., Benítez H.A., Lemic D. (2018). Two decades of invasive western corn rootworm population monitoring in Croatia. Insects.

[101] Williamson M., Fitter A. (1996). The varying success of invaders. Ecology.

[102] Sakai A.K., Allendorf F.W., Holt J.S., Lodge D.M., Molofsky J., With K.A., Baughman S., Cabin R.J., Cohen J.E., Ellstrand N.C. (2001). The population biology of invasive species. Annu. Rev. Ecol. Syst..

[103] Renault D., Laparie M., McCauley S.J., Bonte D. (2018). Environmental adaptations, ecological filtering, and dispersal central to insect invasions. Annu. Rev. Entomol..

[104] Bermond G.B., Li H., Guillemaud T., Toepfer S. (2021). Genetic and phenotypic effects of hybridization in independently introduced populations of the invasive maize pest *Diabrotica virgifera virgifera* in Europe. J. Entomol. Acarol. Res..

[105] Bermond G., Blin A., Vercken E., Ravigné V., Rieux A., Mallez S., Morel-Journel T., Guillemaud T. (2013). Estimation of the dispersal of a major pest of maize by cline analysis of a temporary contact zone between two invasive outbreaks. Mol. Ecol..

[106] Kim K.S., Sappington T.W. (2005). Genetic structuring of western corn rootworm (Coleoptera: Chrysomelidae) populations in the United States based on microsatellite loci analysis. Environ. Entomol..

[107] Kim K.S., Ratcliffe S.T., French B.W., Liu L., Sappington T.W. (2008). Utility of EST-derived SSRs as population genetics markers in a beetle. J. Hered..

[108] Flagel L.E., Bansal R., Kerstetter R.A., Chen M., Carroll M., Flannagan R., Clark T., Goldman B.S., Michel A.P. (2014). Western corn rootworm (*Diabrotica virgifera virgifera*) transcriptome assembly and genomic analysis of population structure. BMC Genom..

[109] Slatkin M. (1987). Gene flow and the geographic structure of natural populations. Science.

[110] Spencer J.L., Hughson S.A., Onstad D.W., Knolhoff L.M. (2023). Insect Resistance Management: Biology, Economics and Prediction.

[111] Onstad D.W., Guse C.A., Crowder D.W., Vidal S., Kuhlmann U., Edwards C.R. (2005). Western Corn Rootworm: Ecology and Management.

[112] Rice M.E. (2018). William B. Showers, Jr.: *Semper Fi* and cutworms that fly. Am. Entomol..

[113] Spencer J.L., Mabry T.R., Levine E., Isard S.A., Vidal S., Kuhlmann U., Edwards C.R. (2005). Western Corn Rootworm: Ecology and Management.

[114] Grant R.H., Seevers K.P. (1989). Local and long-range movement of adult western corn rootworm (Coleoptera: Chrysomelidae) as evidenced by washup along southern Lake Michigan shores. Environ. Entomol..

[115] Isard S.A., Kristovich D.A.R., Gage S.H., Jones C.J., Laird N.F. (2001). Atmospheric motion systems that influence the redistribution and accumulation of insects on the beaches of the Great Lakes in North America. Aerobiologia.

[116] Isard S.A., Spencer J.L., Mabry T.R., Levine E. (2004). Influence of atmospheric conditions on high-elevation flight of western corn rootworm (Coleoptera: Chrysomelidae). Environ. Entomol..

[117] Spencer J.L. (2020). Getting high with the beetles. Am. Entomol..

[118] Johnson C.G., Rainey R.C. (1976). Insect Flight.

[119] Davis M.A. (1980). Why are most insects short fliers?. Evol. Theory.

[120] Coats S.A., Tollefson J.J., Mutchmor J.A. (1986). Study of migratory flight in the western corn rootworm (Coleoptera: Chrysomelidae). Environ. Entomol..

[121] Coats S.A., Mutchmor J.A., Tollefson J.J. (1987). Regulation of migratory flight by juvenile hormone mimic and inhibitor in the western corn rootworm (Coleoptera: Chrysomelidae). Ann. Entomol. Soc. Am..

[122] Naranjo S.E. (1990). Comparative flight behavior of *Diabrotica virgifera virgifera* and *Diabrotica barberi* in the laboratory. Entomol. Exp. Appl..

[123] Naranjo S.E. (1990). Influence of two mass-marking techniques on survival and flight behavior of *Diabrotica virgifera virgifera* (Coleoptera: Chrysomelidae). J. Econ. Entomol..

[124] Stebbing J.A., Meinke L.J., Naranjo S.E., Siegfried B.D., Wright R.J., Chandler L.D. (2005). Flight behavior of methyl-parathion resistant and susceptible western corn rootworm (Coleoptera: Chrysomelidae) populations from Nebraska. J. Econ. Entomol..

[125] Yu E.Y., Gassmann A.J., Sappington T.W. (2019). Effects of larval density on dispersal and fecundity of western corn rootworm, *Diabrotica virgifera virgifera* LeConte (Coleoptera: Chrysomelidae). PLoS One.

[126] Sappington T.W., Showers W.B. (1992). Reproductive maturity, mating status, and long-duration flight behavior of *Agrotis ipsilon* (Lepidoptera: Noctuidae) and the conceptual misuse of the oogenesis-flight syndrome by entomologists. Environ. Entomol..

[127] Zhao X.C., Feng H.Q., Wu B., Wu X.F., Liu Z.F., Wu K.M., McNeil J.N. (2009). Does the onset of sexual maturation terminate the expression of migratory behaviour in moths? A study of the oriental armyworm, *Mythimna separata*. J. Insect Physiol..

[128] Tigreros N., Davidowitz G. (2019). Flight-fecundity tradeoffs in wing-monomorphic insects. Adv. Insect Phys..

[129] Guo J., Yang F., Zhang H., Lin P., Zhai B., Lu Z., Hu G., Liu P. (2022). Reproduction does not impede the stopover departure to ensure a potent migration in *Cnaphalocrocis medinalis* moths. Insect Sci..

[130] Vinal S.C. (1917). The European corn borer, *Pyrausta nubilalis* Hubner, a recently established pest in Massachusetts. Mass. Agr. Expt. Sta. Bull..

[131] Caffrey D., Worthley L. (1927).

[132] Klun J.A., Chapman O.L., Mattes K.C., Wojtkowski P.W., Beroza M., Sonnet P.E. (1973). Insect sex pheromones: minor amount of opposite geometrical isomer critical to attraction. Science.

[133] Kochansky J., Cardé R.T., Liebherr J., Roelofs W.L. (1975). Sex pheromone of the European com borer, *Ostrinia nubilalis*, in New York. J. Chem. Ecol..

[134] Levy R.C., Kozak G.M., Wadsworth C.B., Coates B.S., Dopman E.B. (2015). Explaining the sawtooth: latitudinal periodicity in a circadian gene correlates with shifts in generation number. J. Evol. Biol..

[135] Mason C.E., Rice M.E., DiFonzo C.D., Porter R.P., Sappington T.W., Hunt T.E., Hellmich R.L., Bauté T.S., Andow D.A., Buntin G.D. (2018).

[136] Roelofs W.L., Du J.W., Tang X.H., Robbins P.S., Eckenrode C.J. (1985). Three European corn borer populations in New York based on sex pheromones and voltinism. J. Chem. Ecol..

[137] O'Rourke M.E., Sappington T.W., Fleischer S.J. (2010). Managing resistance to Bt crops in a genetically variable insect herbivore, *Ostrinia nubilalis*. Ecol. Appl..

[138] Dopman E.B., Robbins P.S., Seaman A. (2010). Components of reproductive isolation between North American pheromone strains of the European corn borer. Evolution.

[139] Coates B.S., Johnson H., Kim K.S., Hellmich R.L., Abel C.A., Mason C., Sappington T.W. (2013). Frequency of hybridization between *Ostrinia nubilalis* E- and Z-pheromone races in regions of sympatry within the United States. Ecol. Evol..

[140] Sorenson C.E., Kennedy G.G., Schal C., Walgenbach J.F. (2005). Geographical variation in pheromone response of the European corn borer, *Ostrinia nubilalis* (Lepidoptera: Crambidae), in North Carolina: a 20-y perspective. Environ. Entomol..

[141] Losey J.E., Calvin D.D., Carter M.E., Mason C.E. (2001). Evaluation of noncorn host plants as a refuge in a resistance management program for European corn borer (Lepidoptera: Crambidae) on Bt-corn. Environ. Entomol..

[142] Losey J.E., Carter M.E., Silverman S.A. (2002). The effect of stem diameter on European corn borer behavior and survival: potential consequences for IRM in Bt-corn. Entomol. Exp. Appl..

[143] Lynch R.E. (1980). European corn borer: yield losses in relation to hybrid and stage of corn development. J. Econ. Entomol..

[144] Bode W.M., Calvin D.D. (1990). Yield-loss relationships and economic injury levels for European corn borer (Lepidoptera: Pyralidae) populations infesting Pennsylvania field corn. J. Econ. Entomol..

[145] Dalecky A., Ponsard S., Bailey R.I., Pélissier C., Bourguet D. (2006). Resistance evolution to Bt crops: predispersal mating of European corn borers. PLoS Biol..

[146] Sappington T.W. (2018). Migratory flight of insect pests within a year-round distribution: European corn borer as a case study. J. Integr. Agric..

[147] Mason C.E., Rice M.E., Calvin D.D., Van Duyn J.W., Showers W.B., Hutchison W.D., Witkowski J.F., Higgins R.A., Onstad D.W., Dively G.P. (1996).

[148] Hutchison W.D., Burkness E.C., Mitchell P.D., Moon R.D., Leslie T.W., Fleischer S.J., Abrahamson M., Hamilton K.L., Steffey K.L., Gray M.E. (2010). Areawide suppression of European corn borer with Bt maize reaps savings to non-Bt maize growers. Science.

[149] Dively G.P., Venugopal P.D., Bean D., Whalen J., Holmstrom K., Kuhar T.P., Doughty H.B., Patton T., Cissel W., Hutchison W.D. (2018). Regional pest suppression associated with widespread Bt maize adoption benefits vegetable growers. Proc. Natl. Acad. Sci. USA.

[150] Alstad D.N., Andow D.A. (1995). Managing the evolution of insect resistance to transgenic plants. Science.

[151] Bourguet D., Desquilbet M., Lemarié S. (2005). Regulating insect resistance management: the case of non-Bt corn refuges in the US. J. Environ. Manage..

[152] Kaçar G., Butrón A., Kontogiannatos D., Han P., Peñaflor M.F.G.V., Farinós G.P., Huang F., Hutchison W.D., de Souza B.H.S., Malvar R.A. (2023). Recent trends in management strategies for two major maize borers: *Ostrinia nubilalis* and *Sesamia nonagrioides*. J. Pest. Sci..

[153] Tabashnik B.E., Fabrick J.A., Carrière Y. (2023). Global patterns of insect resistance to transgenic Bt crops: the first 25 years. J. Econ. Entomol..

[154] Smith J.L., Farhan Y. (2023). Monitoring resistance of *Ostrinia nubilalis* (Lepidoptera: Crambidae) in Canada to Cry toxins produced by Bt corn. J. Econ. Entomol..

[155] Smith J.L., Farhan Y., Schaafsma A.W. (2019). Practical resistance of *Ostrinia nubilalis* (Lepidoptera: Crambidae) to Cry1F *Bacillus thuringiensis* maize discovered in Nova Scotia, Canada. Sci. Rep..

[156] Showers W.B., Reed G.L., Robinson J.F., Derozari M.B. (1976). Flight and sexual activity of the European corn borer. Environ. Entomol..

[157] Showers W.B., Berry E.C., Kaster L.V. (1980). Management of 2nd-generation European corn borer by controlling moths outside the cornfield. J. Econ. Entomol..

[158] DeRozari M.B., Showers W.B., Shaw R.H. (1977). Environment and the sexual activity of the European corn borer. Environ. Entomol..

[159] Sappington T.W., Showers W.B. (1983). Adult European corn borer (Lepidoptera: Pyralidae) flight activity in and away from aggregation sites. Environ. Entomol..

[160] Pleasants J.M., Bitzer R.J. (1999). Aggregation sites for adult European corn borers (Lepidoptera: Crambidae): A comparison of prairie and non-native vegetation. Environ. Entomol..

[161] Sappington T.W. (2005). First-flight adult European corn borer (Lepidoptera: Crambidae) distribution in roadside vegetation relative to cropping patterns and corn phenology. Environ. Entomol..

[162] Bailey R.I., Bourguet D., Pallec A.L., Ponsard S. (2007). Dispersal propensity and settling preferences of European corn borers in maize field borders. J. Appl. Ecol..

[163] Hun T.E., Higley L.G., Witkowski J.F., Young L.J., Hellmich R.L. (2001). Dispersal of adult European corn borer (Lepidoptera: Crambidae) within and proximal to irrigated and non-irrigated corn. J. Econ. Entomol..

[164] Qureshi J.A., Buschman L.L., Throne J.E., Ramaswamy S.B. (2005). Adult dispersal of *Ostrinia nubilalis* Hübner (Lepidoptera: Crambidae) and its implications for resistance management in Bt-maize. J. Appl. Entomol..

[165] Merrill S.C., Walter S.M., Peairs F.B., Schleip E.M. (2013). The distribution of European corn borer (Lepidoptera: Crambidae) moths in pivot-irrigated corn. J. Econ. Entomol..

[166] Spangler S.M., Calvin D.D. (2000). Influence of sweet corn growth stages on European corn borer (Lepidoptera: Crambidae) oviposition. Environ. Entomol..

[167] Pilcher C.D., Rice M.E. (2001). Effect of planting dates and *Bacillus thuringiensis* corn on the population dynamics of European corn borer (Lepidoptera: Crambidae). J. Econ. Entomol..

[168] Sappington T.W. (2024). Critical facets of European corn borer adult movement ecology relevant to mitigating field resistance to Bt-corn. Insects.

[169] Heidel W. (2007). The European corn borer in Mecklenburg-Western Pomerania – spreading of the pest and strategies for control. Nachrichtenblatt Dtsch. Pflanzenschutzd..

[170] Chiang H.C. (1961). Fringe populations of the European corn borer, *Pyrausta nubilalis*: their characteristics and problems. Ann. Entomol. Soc. Am..

[171] Chiang H.C. (1972). Dispersion of the European Corn Borer (Lepidoptera: Pyralidae) in Minnesota and South Dakota, 1945 to 1970. Environ. Entomol..

[172] Palmer D.F., Schenk T.C., Chiang H.C. (1985). https://hdl.handle.net/11299/139529.

[173] Chiang H.C., Sisson V., Ewert M.A. (1965). Northerly movement of corn borer moths in southern Minnesota. Proc. Minn. Acad. Sci..

[174] Bretherton R.F., Chalmers-Hunt J.M. (1989). Immigration of Lepidoptera to the British Isles in 1988. Entomol. Record J. Variation.

[175] Colenutt S.R. (1995). *Evergestis limbata* (L.) (Lep: Pyralidae) new to mainland Britain. Entomol. Record J. Variation.

[176] Langmaid J.R., Young M.R. (2006). Microlepidoptera review of 2005. Entomol. Record J. Variation.

[177] Mikkola K., Danthanarayana W. (1986). Insect Flight: Dispersal and Migration.

[178] Pedgley D.E., Yathom S. (1993). Windborne moth migration over the Middle East. Ecol. Entomol..

[179] Showers W.B., Hellmich R.L., Derrick-Robinson M.E., Hendrix W.H. (2001). Aggregation and dispersal behavior of marked and released European corn borer (Lepidoptera: Crambidae) adults. Environ. Entomol..

[180] Reardon B.J., Sumerford D.V., Sappington T.W. (2006). Dispersal of newly-eclosed European corn borer adults (Lepidoptera: Crambidae) from corn into small-grain aggregation plots. J. Econ. Entomol..

[181] Reardon B.J., Sappington T.W. (2007). Effect of age and mating status on adult European corn borer (Lepidoptera: Crambidae) dispersal from small-grain aggregation plots. J. Econ. Entomol..

[182] Dorhout D.L., Sappington T.W., Rice M.E. (2008). Evidence for obligate migratory flight behavior in young European corn borer (Lepidoptera: Crambidae) females. Environ. Entomol..

[183] Dorhout D.L., Sappington T.W., Lewis L.C., Rice M.E. (2011). Flight behaviour of European corn borer infected with *Nosema pyrausta*. J. Appl. Entomol..

[184] Ribak G., Barkan S., Soroker V. (2017). The aerodynamics of flight in an insect flight-mill. PLoS One.

[185] Minter M., Pearson A., Lim K.S., Wilson K., Chapman J.W., Jones C.M. (2018). The tethered flight technique as a tool for studying life-history strategies associated with migration in insects. Ecol. Entomol..

[186] Naranjo S.E. (2019). Assessing insect flight behavior in the laboratory: a primer on flight mill methodology and what can be learned. Ann. Entomol. Soc. Am..

[187] Showers W.B., Weiss M.J., Derrick M.E., Hendrix W.H. (1995). Potential movement on surface airflow of a bivoltine population of European corn borer (Pyralidae: Lepidoptera) into a historically univoltine habitat. Environ. Entomol..

[188] Riley J.R., Reynolds D.R., Smith A.D., Edwards A.S., Zhang X.X., Cheng X.N., Wang H.K., Cheng J.Y., Zhai B.P. (1995). Observations of the autumn migration of the rice leaf roller *Cnaphalocrosis medinalis* (Lepidoptera: Pyralidae) and other moths in eastern China. Bull. Entomol. Res..

[189] Feng H., Wu K., Cheng D., Guo Y. (2004). Spring migration and summer dispersal of *Loxostege sticticalis* (Lepidoptera: Pyralidae) and other insects observed with radar in northern China. Environ. Entomol..

[190] Tollefson J.J., Calvin D.D., Pedigo L.P., Buntin G.D. (1994). Handbook of Sampling Methods for Arthropods in Agriculture.

[191] Pilcher C.D., Rice M.E. (1998). Management of European corn borer (Lepidoptera: Crambidae) and corn rootworms (Coleoptera: Chrysomelidae) with transgenic corn: A survey of farmer perceptions. Am. Entomol..

[192] Crawford H.G., Spencer G.J. (1922). The European corn borer control measures. J. Econ. Entomol..

[193] Felt E.P. (1922). The European corn borer in New York State. J. Econ. Entomol..

[194] Umeozor O.C., Van Duyn J.W., Bradley J.R., Kennedy G.G. (1985). Comparison of the effect of minimum-tillage treatments on the overwintering emergence of European corn borer (Lepidoptera: Pyralidae) in cornfields. J. Econ. Entomol..

[195] Chiang H.C., Hodson A.C. (1972). Population fluctuations of the European corn borer, *Pyrausta nubilalis*, at Waseca, Minnesota, 1948–70. Environ. Entomol..

[196] Showers W.B., DeRozari M.B., Reed G.L., Shaw R.H. (1978). Temperature-related climatic effects on survivorship of the European corn borer. Environ. Entomol..

[197] Krumm J.T., Hunt T.E., Skoda S.R., Hein G.L., Lee D.J., Clark P.L., Foster J.E. (2008). Genetic variability of the European corn borer, *Ostrinia nubilalis*, suggests gene flow between populations in the Midwestern United States. J. Insect Sci..

[198] Kim K.S., Bagley M.J., Coates B.S., Hellmich R.L., Sappington T.W. (2009). Spatial and temporal genetic analyses show high gene flow among European corn borer (Lepidoptera: Crambidae) populations across the central U.S. Corn Belt. Environ. Entomol..

[199] Kim K.S., Coates B.S., Bagley M.J., Hellmich R.L., Sappington T.W. (2011). Genetic structure and gene flow among European corn borer (Lepidoptera: Crambidae) populations from the Great Plains to the Appalachians of North America. Agric. For. Entomol..

[200] Coates B.S., Kozak G.M., Kim K.S., Sun J., Wang Y., Fleischer S.J., Dopman E.B., Sappington T.W. (2019). Influence of host plant, geography and pheromone strain on genomic differentiation in sympatric populations of *Ostrinia nubilalis*. Mol. Ecol..

[201] Bourguet D., Bethenod M.T., Pasteur N., Viard F. (2000). Gene flow in the European corn borer *Ostrinia nubilalis*: implications for the sustainability of transgenic insecticidal maize. Proc. Biol. Sci..

[202] Martel C., Réjasse A., Rousset F., Bethenod M.T., Bourguet D. (2003). Host-plant-associated genetic differentiation in northern French populations of the European corn borer. Heredity.

[203] Leniaud L., Audiot P., Bourguet D., Frérot B., Genestier G., Lee S.F., Malausa T., Le Pallec A.H., Souqual M.C., Ponsard S. (2006). Genetic structure of European and Mediterranean maize borer populations on several wild and cultivated host plants. Entomol. Exp. Appl..

[204] Malausa T., Dalecky A., Ponsard S., Audiot P., Streiff R., Chaval Y., Bourguet D. (2007). Genetic structure and gene flow in French populations of two *Ostrinia* taxa: host races or sibling species?. Mol. Ecol..

[205] Frolov A.N., Audiot P., Bourguet D., Kononchuk A.G., Malysh J.M., Ponsard S., Streiff R., Tokarev Y.S. (2012). From Russia with lobe: Genetic differentiation in trilobed uncus *Ostrinia* spp. follows food plant, not hairy legs. Heredity.

[206] Wright S. (1946). Isolation by distance under diverse systems of mating. Genetics.

[207] Wright S. (1969).

[208] Rousset F. (1997). Genetic differentiation and estimation of gene flow from F-statistics under isolation by distance. Genetics.

[209] Shirk A.J., Cushman S.A. (2014). Spatially-explicit estimation of Wright’s neighborhood size in continuous populations. Front. Ecol. Evol..

[210] Sappington T.W., Spencer J.L. (2023). Movement ecology of adult western corn rootworm: implications for management. Insects.

[211] Reid C. (1899).

[212] Clark J.S., Fastie C., Hurtt G., Jackson S.T., Johnson C., King G.A., Lewis M., Lynch J., Pacala S., Prentice C. (1998). Reid’s paradox of rapid plant migration: dispersal theory and interpretation of paleoecological records. Bioscience.

[213] Caswell H., Lensink R., Neubert M.G. (2003). Demography and dispersal: life table response experiments for invasion speed. Ecology.

[214] Marko P.B. (2004). ‘What’s larvae got to do with it?’ Disparate patterns of post-glacial population structure in two benthic marine gastropods with identical dispersal potential. Mol. Ecol..

[215] Jones A. (2010). Reconciling field observations of dispersal with estimates of gene flow. Mol. Ecol..

[216] Yu H., Nason J.D., Ge X., Zeng J. (2010). Slatkin’s Paradox: when direct observation and realized gene flow disagree. A case study in *Ficus*. Mol. Ecol..

[217] Harnik P.G., Maherali H., Miller J.H., Manos P.S. (2018). Geographic range velocity and its association with phylogeny and life history traits in North American woody plants. Ecol. Evol..

[218] Huestis D.L., Dao A., Diallo M., Sanogo Z.L., Samake D., Yaro A.S., Ousman Y., Linton Y.-M., Krishna A., Veru L. (2019). Windborne long-distance migration of malaria mosquitoes in the Sahel. Nature.

[219] Florio J., Verú L.M., Dao A., Yaro A.S., Diallo M., Sanogo Z.L., Samaké D., Huestis D.L., Yossi O., Talamas E. (2020). Diversity, dynamics, direction, and magnitude of high-altitude migrating insects in the Sahel. Sci. Rep..

[220] Hallatschek O., Fisher D.S. (2014). Acceleration of evolutionary spread by long-range dispersal. Proc. Natl. Acad. Sci. USA.

[221] Steyn V.M., Mitchell K.A., Terblanche J.S. (2016). Dispersal propensity, but not flight performance, explains variation in dispersal ability. Proc. Biol. Sci..

[222] Caton B.P., Fang H., Manoukis N.C., Pallipparambil G.R. (2021). Quantifying insect dispersal distances from trapping detections data to predict delimiting survey radii. J. Appl. Entomol..

[223] Liu B.R. (2021). Biphasic range expansions with short- and long-distance dispersal. Theor. Ecol..

[224] Schneider C. (2003). The influence of spatial scale on quantifying insect dispersal: An analysis of butterfly data. Ecol. Entomol..

[225] Reynolds D.R., Chapman J.W., Harrington R. (2006). The migration of insect vectors of plant and animal viruses. Adv. Virus Res..

[226] Byrne D.N., Rathman R.J., Orum T.V., Palumbo J.C. (1996). Localized migration and dispersal by the sweet potato whitefly, *Bemisia tabaci*. Oecologia.

[227] Spurgeon D.W., Suh C.P.-C. (2019). Termination of diapause in the boll weevil (Coleoptera: Curculionidae). J. Econ. Entomol..

[228] Culin J., Brown S., Rogers J., Scarborough D., Swift A., Cotterill B., Kovach J. (1990). A simulation model examining boll weevil dispersal: historical and current situations. Environ. Entomol..

[229] Kim K.S., Jones G.D., Westbrook J.K., Sappington T.W. (2010). Multidisciplinary fingerprints: forensic reconstruction of an insect reinvasion. J. R. Soc. Interface.

[230] Westbrook J.K., Eyster R.S., Allen C.T. (2011). A model for long-distance dispersal of boll weevils (Coleoptera: Curculionidae). Int. J. Biometeorol..

[231] Johnson W.L., Cross W.H., McGovern W.L. (1976). Long-range dispersal of marked boll weevils in Mississippi during 1974. Ann. Entomol. Soc. Am..

[232] Guerra A.A. (1988). Seasonal boll weevil movement between northeastern Mexico and the Rio Grande Valley of Texas, USA. Southwest. Entomol..

[233] Jones R.W., Cate J.R., Martinez Mernandez E., Treviño Navarro R. (1992). Hosts and seasonal activity of the boll weevil (Coleoptera: Curculionidae) in tropical and subtropical habitats of northeastern Mexico. J. Econ. Entomol..

[234] Lukefahr M.J., Barbosa S., Sabrinho R.B. (1994). The introduction and spread of the boll weevil, *Anthonomus grandis* Boh. (Coleoptera) in Brazil. Southwest. Entomol..

[235] Spurgeon D.W., Raulston J.R., Zamora O.Z., Loera J. (1997). Spatial and temporal patterns of boll weevil trap captures in northeastern Mexico. Proc. Beltwide Cotton Conf..

[236] Glick P.A. (1939).

[237] Rummel D.R., Jordan L.B., White J.R., Wade L.J. (1977). Seasonal variation in the height of boll weevil flight. Environ. Entomol..

[238] Kim K.S., Sappington T.W. (2013). Population genetics strategies to characterize long-distance dispersal of insects. J. Asia Pac. Entomol..

[239] Alvarado A., Jones R.W., Pedraza-Lara C., Alvarado Villanueva O., Pfeiler E. (2017). Reassessment of the phylogeography and intraspecific relationships of western and eastern populations of the boll weevil, *Anthonomus grandis* Boheman (Coleoptera: Curculionidae), in North America. Biol. J. Linn. Soc..

[240] Kim K.S., Cano-Ríos P., Sappington T.W. (2006). Using genetic markers and population assignment techniques to infer origin of boll weevils (Coleoptera: Curculionidae) unexpectedly captured near an eradication zone in Mexico. Environ. Entomol..

[241] Kim K.S., Sappington T.W., Allen C.T. (2008). Genetic profiling to determine potential origins of boll weevils (Coleoptera: Curculionidae) captured in a Texas eradication zone: endemicity, immigration, or sabotage?. J. Econ. Entomol..

[242] Cesari M., Maistrello L., Piemontese L., Bonini R., Dioli P., Lee W., Park C.-G., Partsinevelos G.K., Rebecchi L., Guidetti R. (2018). Genetic diversity of the brown marmorated stink bug *Halyomorpha halys* in the invaded territories of Europe and its patterns of diffusion in Italy. Biol. Invasions.

[243] Leskey T.C., Nielsen A.L. (2018). Impact of the invasive brown marmorated stink bug in North America and Europe: history, biology, ecology, and management. Annu. Rev. Entomol..

[244] Jackson P.L., Straussfogel D., Lindgren B.S., Mitchell S., Murphy B. (2008). Radar observation and aerial capture of mountain pine beetle, *Dendroctonus ponderosae* Hopk. (Coleoptera: Scolytidae) in flight above the forest canopy. Can. J. For. Res..

[245] Chen H., Jackson P.L. (2017). Climatic conditions for emergence and flight of mountain pine beetle: implications for long-distance dispersal. Can. J. For. Res..

[246] Rovnyak A.M., Burks C.S., Gassmann A.J., Sappington T.W. (2018). Interrelation of mating, flight, and fecundity in navel orangeworm females. Entomol. Exp. Appl..

